# On Insanity, Considered in a Pathological, Philosophical, Historical, and Judicial Point of View, from the Revival of Learning in Europe until the Nineteenth Century

**Published:** 1846-07

**Authors:** 


I. De la Folie, considérée sous le Point de vue Pathologique, Philosophique, &c. &c. Par *L. F. Calmeil*, D.M P. Paris 1845.

On Insanity, considered in a Pathological, Philosophical, Historical, and Judicial point of view, from the Revival of Learning in Europe until the Nineteenth Century. By *L. F. Calmeil*, Physician of Charenton. Two Vols. 8vo. pp. 1050. Paris, 1845.

II. An Act (8 & 9 Vic. c. 100) for the Regulation of the Care and Treatment of Lunatics. With Explanatory Notes and Comments, &c. Edited by *G. Forbes Winslow*, M.D. 12mo. pp. 173. London, 1845.

III. Observations and Essays on the Statistics of Insanity. To which are added the Statistics of the Retreat, near York. By *John Thurnam*, M.D. 8vo. pp. 350. London, 1845.

IV. The American Journal of Insanity. October 1845.

V. A Letter to Lord Ashley, M.P. By *W. B. Costello*, M.D. 1846.

VI. Clinical Lectures on the Principal Forms of Insanity. By *John Conolly*, M.D. (Publishing in the Lancet).

VII. Annual Report of the Managers of the New York State Lunatic Asylum. By *Amariah Brigham*, M.D. 1845.

The above publications treat of the past, present, and future condition of the Insane, upon each of which points we are desirous of offering a few remarks. It would be surprising indeed, if, amid the now generally prevalent disposition to search out for the causes and means of relieving the social, moral, and physical evils which afflict the unfortunate, the cause of the Lunatic, the most helpless of all sufferers, were forgotten. That happily this is not the case the labours and devotion of so many highly-gifted individuals throughout Europe and America, the progressive amelioration of his condition, the warm sympathy with which the public has watched the various attempts at accomplishing this, and the readiness with which our Government and Legislature have so recently accorded most extensive means for future improvements, combine to prove.

The history of the management of the insane presents but a humiliating picture of human intelligence and progress. It does not seem that any provision for even their safe-keeping existed prior to the Reformation, and long after the establishment of "Bedlam," indeed to comparatively modern times, the more harmless were allowed to roam about, objects of the jest, contumely, and cruel treatment of the thoughtless and ignorant; while such as manifested dispositions for violence or insubordination were submitted to a system of management, the sole rule of which was domination by brute force, and its sole result the production of an enormous amount of the most terrible suffering. Yet did not all this arise from any indisposition to succour the afflicted; for we find, as one of the natural and earliest results of Christianity, the establishment of hospitals for the sick and wounded; and the vast numbers and great resources of some of those devoted to the cure of leprosy in the middle ages prove to us that our ancestors were in no-wise wanting in beneficence, when a mode of exercising it was indicated to them. That there were no establishments at an earlier period for the treatment of the insane arose from the fact of the nature of the malady being misunderstood, and no doubt in some measure from a far less proportionate and absolute number of persons being the subject of it than in modern times; for that the number of persons liable to insanity, and the varieties of the disease, have increased in modern times, and as a consequence of the highly-wrought and somewhat artificial condition of modern society, cannot be doubted. But our forefathers failed in recognising the malady in numerous instances in which it presented itself. M. Calmeil's Treatise on Insanity is almost entirely occupied in detailing instances in which symptoms that would be clearly recognized in our day as insane delusions were believed to result from demoniacal possessions and sorcery. We must confess that prior to the perusal of this work we had no idea of the extent to which these erroneous views have prevailed on the Continent. The accusations of sorcery and witchcraft were tolerably numerous during the dark periods of our own history: but the wholesale denunciations and punishments recorded in M. Calmeil's work infinitely exceed these. During the 15th and 16th centuries, ten, twenty, or more than a hundred such persons at a time were handed over to be dealt with by the law; and in one reign alone, that of Francis 1st. (1515-47), 100,000 persons were destroyed or otherwise punished on this account. The class of persons, likewise, who endured these persecutions was different. In this country they were mostly wretched old women whose ugliness or eccentricity had rendered them remarkable; on the Continent they were usually members of some of the numerous religious convents. Numbers of women, submitted to the austerities of these abodes, became attacked with perverted religious ideas, and frequently by convulsive movements and various other hysterical symptoms. They confessed and gloried in the most revolting accounts of their profanation of the religion they had sworn to observe, as also their intercourse with and obedience to diabolical agencies. Exorcism was the remedy resorted to. Priests and bishops devoted days and nights to the employment of every known mode of expulsion; and usually, so far from any benefit accruing, the consequence was, that the disease became contagious in the convent and not infrequently epidemic in the district; so that months or years might elapse ere a decent tranquillity were restored. During their paroxysms these poor women, hitherto of irreproachable lives, confessed to the greatest enormities, and did not hesitate to accuse their dearest friends or venerated superiors as participators or originators of these. The stake was put into requisition on every side, and hundreds and thousands of poor creatures perished the victims of their own fancies or of the delusions of others. On some occasions, persons falsely accused, excited by the religious ceremonies they were subjected to, eventually acknowledged all the enormities attributed to them, and even exaggerated these; while the very priests engaged in the exorcisms, occasionally became themselves afterwards the subjects of similar delusions. M. Calmeil reviews the particulars of all the considerable epidemics of this kind on record at great length, and justly observes that the same delusions are to be observed among our insane at the present day. Much more infrequently however, for every epoch of history may almost be said to have its delusion characteristic of the condition of society at the time. M. Calmeil thus expresses himself upon this point.

"It has been proved for this last half century that insanity is liable to take its colour from the religious persuasions, the philosophical or superstitious ideas, or the social prejudices which happen to be actually in vogue; that this colour varies in the same country according to its political circumstances, civil commotions, literary productions, theatrical representations, or the description, progress and direction of its industry, arts and sciences. Thus, since elementary ideas of physics and chemistry have become to some extent popular among us, we find many of the visionaires disreason in our large towns concerning electricity, balloons, burning-glasses, telegraphs, air-guns, and optical experiments. What numbers since the first experiments of Mesmer have complained of being victims of the persecutions of the magnetisers and somnambulists. Long after the Reign of Terror had ceased to oppress France, the unfortunate persons who, during the storms of 1793, had felt the danger of oppression, and whose reason had afterwards lost its equilibrium, trembled anew for property and riches, for the preservation of their lives, and for the safety of their relatives and friends. On the contrary, those of the insane who had taken an active part in the incendiary movements of that terrible epoch, vividly exhibited their fears of the resentment of the clergy and nobility. Under the reign of Napoleon, insanity had its heroes who employed themselves in giving commands, as if they had whole armies under their direction. After the fall of the glories of the Empire, when the remembrance of our disasters, reverses, and deceptions were rankling in the hearts of all true citizens, the lypemaniacs were apprehending new invasions, cruelties, and insolence on the parts of the conquerors and the Cossacks. In our own day, the melancholics are affrighted at the soldiery, the police, the jury, the courts, the guillotine and the galleys. At all periods, the expression of insanity has undergone analagous variations. When we have once acquired a knowledge of the nature of the ideas, sentiments, and passions which are fermenting in the bosoms of societies and families, so that we are enabled to appreciate the intellectual and moral condition of the masses at different historical periods, we almost know by anticipation in what direction insane ideas must lean in the different phases of civilization.

"In the 15th century, insanity especially bore the impressions of the superstitious ideas and theological doctrines then in repute. It was necessarily so. These doctrines had been exhibited, developed and defended in the schools, taught in the religious houses, explained to every one from the pulpits, and amply commented upon to all the faithful at the confessional. Persons who then lost their reason almost always erred in ideas or sensations relative to demons, angels, supernatural beings, just because such subjects were familiar to them, and had made a deep impression upon their minds and imaginations. It is certain that the inquisitors, who in many places performed the functions of judges, accepted the most improbable and atrocious assertions. They, indeed, went much farther than this, for they often obliged the insane to unfold the symptoms of their disease in the midst of torture, and afterwards sent them to finish their lives at the stake. The facts we shall advert to will exhibit to us the different shades of delirium which were mistaken by our ancestors. Every one now allows that the possessed, the lycanthropes, and the demoniacs were the subjects of a morbid condition; and how must we pity so many unfortunate creatures, when an undiscovered disease led them to declare that they were in accord with the devil, to curse their Creator, to outrage Nature and Providence, and trample under foot all that should inspire man with respect and veneration? But how can we also refuse some indulgence to those who were charged with the preservation of morals and religion and the execution of the laws, when it is evident that ignorance as much as fanaticism contributed to close their hearts to pity?"

In reference to this latter point we must remember, before we charge these persons with cruelty rather than ignorance, that the belief in the power of supernatural agents was not a mere vulgar credence, but one held almost universally, and that even by persons of the strongest minds, as indeed is amply proved when we cite the names of Luther and Melancthon. Moreover, although in the 15th century ecclesiastics were almost the only persons who interfered in the management, relief, and punishment of persons supposed to be possessed, and doubtless by their exhortations, threats, and imposing public ceremonials, increased the manifestations of disease which might have been relieved by segregation and medicinal appliances—yet, even in the following century, men, bearing the important names of Ambroise Paré, Fernel, J. B. Porta, and Cardan, did not hesitate to refer such symptoms to demoniacal agency, and did not suggest more rational procedures for their removal.

We have not space to pursue the consideration of any of the numerous narrations brought under notice by M. Calmeil, whose work, as a minute and laborious record of the various insane delusions which have manifested themselves during the 15th, 16th, 17th and 18th centuries, will prove as valuable an aid to future historians of the human mind as Hecker's History of the various other epidemic visitations of the Middle Ages. For the medical enquirer the book also possesses some additional interest, from the author prefacing the history of the delusions of each century with an account of the ideas on mental pathology entertained by the leading men of the epoch—tracing them from the purely theological views which exclusively prevailed in the 15th century to the juster ones which immediately preceded those enounced by Pinel in the 18th. In this way, the opinions of Bonet, Boerhaave, Morgagni, Lorry, Bayle, Sauvages, Senertius, and especially those of our countryman Thomas Willis, are analysed at considerable length. The histories of the extato-convulsive epidemics prevailing among the persecuted Huguenots and Jansenists in the 18th century are detailed with great minuteness; and those who are now staring and gaping at the wonderments of modern mesmerism may find in these narrations the prototypes of every pretension, and marvels as regards the endurance of physical suffering not yet attempted to be rivalled. M. Calmeil also enters into a detailed refutation of the pretensions of Mesmer.

If an ignorance of the pathological condition of their victims may be urged in extenuation of the cruelties practised upon them by inquisitors and exorcists, an equal ignorance of the most appropriate means of relieving or assuaging the consequences of such state prevailed long after its existence had been explicitly acknowledged. Fear was the only passion sought to be wrought upon, and no engine for its excitement was deemed too harsh in its operation; so that the condition of the helpless lunatic, as regards positive suffering, during the last century and commencement of the present, was more hapless than that of the prisoner when prisons were such as Howard has depicted them. When we find Cullen and other enlightened and humane practitioners sanctioning the use of blows and stripes as a curative measure, we may easily imagine the dreadful cruelties which must have been perpetrated when the administration of these and of other coercive means was entrusted to brutal and ignorant persons. But a far graver reproach than that of ignorance, namely, apathy, has, until now, attached to our own country. Who could have believed that, so long after the grand experiments of Pinel in France and the Quakers in England had demonstrated the feasibility and superiority of a humane mode of treatment, the labours of Esquirol in the one country, and of Parliamentary Committees in the other, should have been enabled to expose to light the revolting abuses they laid open? Nay, at this very time, more than half-a-century after the above epoch, a large portion of which has been passed in profound peace, and with every circumstance favourable to the diffusion of the knowledge of improvement, the Metropolitan Commissioners have found poor wretches chained and ironed and wallowing in their excrement and filth, uncared for and unseen; while Dr. Conolly saw a patient at Rouen, who had been tightly confined in a straw crib for nine years! The perusal of the history of the Retreat near York, as detailed by Dr. Thurnam, gives rise at once to the feelings of pride and shame: pride, that so noble and so successful an experiment should have originated in our country; and shame, that so little practical good has resulted to the mass of the insane from its institution. It is true that the Retreat was visited from all quarters at home and abroad, and that its success shamed the governors of the York Asylum into an enquiry and redress of the dreadful grievances of that establishment: but the disclosures of the Committee of the House of Commons in 1815, eighteen years after the opening of the Retreat, and again in 1828, too plainly exhibited how little general practical benefit had resulted from the example. Various legislative measures have been from time to time passed, as some glaring abuse has come to light, but none of so extensive and comprehensive a character as Lord Ashley's Act, just come into operation, and passed in consequence of the representations of the Metropolitan Commissioners, whose Report we reviewed some time since.*

The object of Dr. F. Winslow's little work is to give an account of the provisions of this new Act; and all those having any thing to do with the insane will find it a most useful companion. We will advert to a few of the most important provisions of the new measure before we state our opinion of its probable operation, and of the *desiderata* it yet leaves unprovided for. In the first place, an amended system of *inspection* has been organized, without which indeed all other improvements would have been nugatory. The Commissioners are to be a well-paid and permanent body, possessing the most extensive powers for supervision and redress of grievances. Why three of the six are to be barristers it would be some-

---

* See Med.-Chir. Review, Jan. 1845, p. 115.

what difficult to assign a reason, except that the members of the long robe have enjoyed a monopoly of government appointments from time immemorial—so that the conceding even a moiety to our profession is very acceptable, if it be only for the novelty of the thing. All who are acquainted with the cruel and neglectful treatment which unfortunate lunatics have received at the hands of their keepers in private lodgings, cottages, &c., will rejoice to learn that henceforth this class of persons is to be submitted to licensing and inspection. Their exemption hitherto from this, and the consequent impunity of their proceedings, however nefarious, has long been a disgrace to our age and country; and—

"If this were the only amendment effected in the law, Lord Ashley would be entitled to the gratitude of every humane mind for having introduced it. * * * Independently of the horrible cruelties to which these unfortunate persons were of necessity exposed, was it possible, I ask, under these unfavourable circumstances, to carry out successfully anything like a successful or efficient plan of medical treatment? Little or nothing could be done to re-establish mental health. Shut out from society, pent up like a wild beast in a small ill-ventilated apartment, left to the exclusive surveillance of a person whose existence, perhaps, depended upon the party continuing in a state of derangement, no prospect of cure could be entertained. As far as *data* will enable us to form a judgment, it has been satisfactorily established that the ratio of cures in cases of insanity confined in unlicensed houses, only amounted to 10 per cent., whilst in licensed it averaged 40. This is a startling fact, and ought at once to awaken the attention of those whose friends or relations are exposed to these disadvantageous results."—*Winslow*, p. 38.

Well might Lord Ashley declare that, if the calamity of insanity overtook him, he should wish to be placed in a public asylum. Wealth in these dreadful cases, instead of as usual procuring an assuagement of evil, is an inducement to its augmentation. In this point of view we entirely disapprove of "persons deriving no profit" from the charge they have undertaken being exempted from the provisions of this Act. The sound policy of the following dictum of Dr. Conolly seems to us unquestionable. "Every insane person should in some way or other become the public care, and be looked after by responsible officers of the state, so as to ensure the hope of recovery and liberty to every curable case and proper protection to all." The exception opens a door to evasions, and the history of the insane proves to us there may be many conceivable cases in which justice may not be done the lunatic even by his own friends and relatives.

The new Act, likewise, enforces a particularization of the special facts which have led to the signature of the certificate consigning the individual to duress. A very proper provision, and one, we have reason to know, is not uncalled for, medical men having wilfully or carelessly signed certificates which they must have withheld, had a detail of their reasons been demanded of them. We have never, however, been enabled to perceive the necessity of two certificates being required. In the great majority of cases one practitioner alone has had the opportunity of observing the patient with sufficient attention to enable him to sign the document in an obscure case, and the second signature has then often been obtained as a mere matter of form, or after injurious delay. The difficulty of obtaining it will be now still greater; for the cases are numerous in which a second practitioner, a stranger to the patient, may be unable to specify the circumstances of an aberration of intellect which may nevertheless exist.

In the third place, if the keepers of private asylums are to be submitted to more active inspection, (which indeed if they know their own true interests will be courted by them, as it will be found eminently instrumental in increasing the reputation of well-conducted establishments) so will they be protected against vexatious law proceedings for false imprisonment in the case of alleged lunatics—they having taken the precautions required by the Act.

Besides the Bill, so ably analysed and commented upon by Dr. Winslow, Lord Ashley introduced another, having for its object the supplying additional accommodation, by compelling the various counties to provide asylums for their poor. A former Act conferred upon them the *power* of so doing without, however, insisting upon their putting it into force. How far they have done so may be judged from the following extract from Lord Ashley's Speech.

"I feel it necessary to call the attention of the House to the principal defects which are pointed out by our Report as to Pauper Lunatics and County Asylums. 1st. That there are 40 counties in England, and only 16 county asylums, and 12 counties in Wales, and only one disgraceful borough asylum. Of the 24 counties having no asylums, one has 500; two upwards of 400; three upwards of 300; seven upwards of 200; and eleven nearly 100 lunatics each; and Wales has 1000 lunatics. The second defect is that of the 16 counties which have asylums, one has 800, one has 600, one has 500, one more than 300, three more than 200, and the rest more than 100 lunatics, for whom there is no accommodation in the asylums that have been erected, and no other receptacle. The third defect is, that all the existing asylums are full of incurables (of 984 patients in Hanwell, only 30 were reported as curable; and only 20 of 382 confined in the Surrey Asylum), or persons said to be incurable. The fourth defect is, that no system has been adopted in county asylums to give preference to urgent cases, or those capable of cure. The fifth defect is the detention of lunatics in workhouses, where there is no sufficient medical or moral treatment. The sixth is, there is no real visitation or true account of those lunatics who are not in asylums; for example, the lunatics of North and South Wales, and those in England not in asylums, being 9,339 with their friends or in workhouses."

Sir James Graham properly observed, after listening to these facts, and to the detail of some of the horrors resulting from their existence, that "the time, I think, has arrived when what was permissive shall be compulsory—when the counties throughout England and Wales shall be compelled by law to find sufficient means and accommodation for the care and custody of those unhappy persons." The creation of the new asylums has therefore been resolved upon, as also of other receptacles for incurable, or to use the more proper appellation employed by Lord Ashley, *chronic* cases. This last provision has been much disapproved of by many practitioners conversant with the management of the insane, and especially by Dr. Conolly, who believes by any such arrangement the interests and comforts of the old cases will be sacrificed. With all deference to so deservedly a high authority, we can scarcely believe this will be the case. Nothing can be worse than the present state of affairs. The asylums, expensively furnished with every curative appliance, are choked up with cases in which these are of no avail, while hundreds and thousands awaiting their turn for admission, which had this been promptly accorded to them would have furnished a large proportion of cures, fall into a hopeless condition in consequence of the delay. They never have their fair chance of cure offered them, and in this way an immense amount of needless suffering and expense is engendered. We are glad to find the change meets with the approval of so experienced an observer as Dr. Thurnam, who also offers some useful suggestions respecting it.

"The proposed plan of erecting asylums for the *care* of the decidedly incureable and comparatively harmless in addition to hospitals for the *cure* and care of other classes of the insane, appears to me to be worthy of every encouragement. The most desirable plan would seem to be that of making such asylums appendages to the hospitals, and of placing their internal government in subordination to the directing physicians of the latter. An assistant physician or other medical officer should be appointed as the resident head. Such asylums should, when possible, be within a quarter or half a mile from the hospitals with which they are connected. Their construction will properly be more simple, the officers and servants less numerous, and their general economy altogether less costly than that of their sister establishments, the hospitals. The formation of such divided but inter-dependent establishments, as that of hospitals and asylums united with each other under a common external and united internal government, will, whenever carried out, constitute an important era in the public provision for the insane poor of these kingdoms; and will, I believe, be found, not only more economical than our county asylums as at present conducted, but, by affording greater facilities for the admission of recent cases into hospitals, also to result in a larger proportion of recoveries and a diminished mortality." 81.

Dr. Conolly observes that, many of the incurables form most eligible attendants upon other patients; and that, if confined by themselves, they would be far less happy and become more and more neglected in proportion as their incurability became better established. The retention of a few such persons as aids to the servants might be advisable, although we cannot but think that in the treatment of curable patients their intercourse with persons of sane mind should be preferred wherever possible. In regard to the neglected condition of the incurable or chronic and epileptic insane we can see no reason whatever why it should take place, and it would imply a want of inspection, and an omission of appliances, certainly not contemplated by the originators of the change. Of course the arbitrary separation of cases into curable and incurable is out of the question; but the adaptation of different establishments to the requirements of various classes of patients seems to us feasible and desirable.

Allowing as we do that the changes in the law effected by Lord Ashley are very great improvements, and perhaps as extensive as the present state of opinion will admit, we may still state our belief that they come very far short of the requirements of the case to be provided for. We believe the system to be radically bad that admits of the care of the lunatic being made an object of private speculation and emolument. His condition is so peculiar that it fully warrants a departure from the ordinary mode of proceeding in this country, and *that he should become, whatever his condition in society, rich or poor, an object of the special and direct management of the State.* That humanity and economy alike demand this change in respect to the *poor* is apparent from the apathy with which local bodies have allowed their wants to remain so long unprovided for, and the disgraceful jobbing which too often takes place when money has to be expended in their relief, so that the results are very inadequate to the outlay. In what other mode are uniformity of management and the rapid spread of improvement in it to be secured? Is it not notorious that the magistrates and governors in some counties most disgracefully neglect the duties of inspection, and in others, unwarrantably interfere with the details of treatment, and impose regulations which are quite inconsistent with the true welfare of the inmates of the establishments under their controul? Surely the nature of the management of lunatics should not depend upon the chance of selection of a body of efficient persons from a class in which the most erroneous views concerning all medical matters so freely prevail. One general, comprehensive system is required.

But we believe State interference is also desirable in the case of the *rich lunatic*. His case is indeed a peculiar one: for, it is a lamentable fact that, when he has once lapsed into this unfortunate condition, his friends, too often regarding his calamity almost in the light of a stigma on the family, or even occasionally profiting by his mischance, do not always evince an over-weaning anxiety for his restoration to society. This may not be frequently the case, and may, and sometimes is, counteracted by the high-mindedness of the principal of the establishment in which the lunatic may be located; but we hold that, where even exceptive instances exist, and where such temptations not to favour a cure may influence many meaner minds, the State should step in and rescue her unfortunate citizen from his predicament. Moreover, with every disposition to do justice to their inmates the proprietors of many, very many, asylums, especially in large towns, are not possessed of space and appliances adequate to the efficient treatment of the insane—their safe custody being too often all that is accomplished. It may be said that a vigilant inspection will counteract the evils now adverted to, and so it will some of them: but the difficulty of organizing a truly effective system of inspection is great beyond conception, if not insuperable. The visitors can only come occasionally, and although they may check flagrant delinquencies, none but residents within the very walls of the asylum itself can ensure the due enforcement of the best contrived modes of management. We believe, then, that asylums for both rich and poor lunatics should be under the immediate direction of Government, and *managed by well-paid, responsible, resident, medical officers*, who could have no interest whatever but in the speedy recovery or the amelioration of the condition of those committed to their care. Moreover, there is a class of persons between the two extremes we have mentioned whose means are limited, yet position in society respectable, and for whom the occurrence of insanity now, owing to the high charges at private asylums, is a sentence of degradation to pauperism, with all its accompanying obstacles to cure. Such persons might be provided for in some such establishments as we have been contemplating, with an attention to their prior habits and present comforts, at charges which could not prove remunerative to private speculation.

So frequent have the instances of sane persons unjustly confined in asylums been brought before the public, that a main object of legislation has been, of late years, the prevention of this enormous abuse. In effecting this, however, it seems to have run into the opposite extreme, and imposed a *veto* upon the admission of persons who would be benefited by residence within the walls of an asylum. We have now a patient exhibiting every symptom of threatened insanity whose condition we have no doubt would be rendered far more favourable by temporary seclusion, and yet, under the present state of the law, no asylum could admit him. We do not doubt that many cases might be checked in the bud by a prompt resort to appropriate measures, among which, separation from accustomed associations is one of the most important. Vigilant inspection, or above all the system of asylum-management above adverted to, would prevent the possibility of such persons being improperly detained, if even inadvertently admitted. Dr. Costello's pamphlet contains some useful observations on this head.

"As the law stands at present, retirement into an asylum is interdicted to those who would repair to them voluntarily, nay cheerfully, and who, it must be acknowledged, could not take any step, under such critical circumstances, better calculated to restore mental health and peace. But a nervous person cannot be admitted to share the advantages best suited to his condition, without the certificates of two medical men that he is of unsound mind. The law has no sympathy for mere apprehensions: it turns a deaf ear to the *egrotare timeus*: he must allow himself to get worse, before the law will allow him to resort to the best means of getting better; then, and not till then does it relent, and shew pity to the victim of its delay. * * * * * The class of persons now referred to, having a full knowledge of what they do, ought to be enabled to resort to asylums of *their own free will and accord,* to avail themselves of the advantages so suited to their infirmities. They require modified isolation, without separation from the world, and where can these conditions be better united, than in a well-conducted asylum, adapted, as it should be, to the treatment of disorders of the mind, even in their mildest forms? If a sufficient security against abuse were provided, and this could be easily accomplished, there could be no valid reason for refusing those benefits to nervous patients as such. But those benefits, important as they are, would not be the sole ones resulting from a change of the law. The very character of the asylum would be changed. From a prison, which it is now so universally regarded, it would become an hospital, and those prejudices which now operate so extensively against persons attacked with insanity would disappear."

These, and other remarks to the same purport, seem to us dictated by sound sense. Every one is aware how important, in a curative point of view, it is that the treatment of a case of insanity should be commenced as early as possible: and yet, until some overt act has been committed, perhaps months after the premonitory symptoms, or sufficient derangement has manifested itself to convince two medical observers of its existence, treatment does not commence.

### Statistics of Insanity.

The dangers of the indiscriminate use of the Numerical System are nowhere brought to light stronger than in the Statistics of Insanity. The accumulation of large masses of undigested figures has given rise to conclusions which a scrutinizing examination of some of their particulars will by no means warrant.

Dr. Thurnam has exhibited more than one generally prevalent error derivable from such hasty generalization. The first chapter of his work is entitled, "*On the Methods of deducing and exhibiting the Results of Treatment; and on the Circumstances in the Character of the Cases admitted, capable of influencing these Results.*" He points out the uncertainty and errors which must result from a want of uniformity in the employment by different observers of such terms as "recovered," "improved," "cured;" and states that, in some establishments, as the York Asylum, prior to 1814, the returns have been purposely falsified. For want of attendance to these and various other particulars, to be afterwards noted, very erroneous and unjust conclusions have been frequently drawn as regards the comparative merits of different establishments.

1. *Calculating the Proportion of Recoveries and Mortality.*—For this, different plans have been adopted. Dr. Thurnam calculates the per-centage of *recoveries* upon the *admissions*, while Mr. Farr calculates it upon the *discharges*, stating that, "if the mortality continued the same, the probability is, that the patients to *be discharged* would, *cæteris paribus*, be discharged cured, relieved, and dead, in the same proportions to those already discharged." To this strange statement Dr. T. demurs, seeing that the recoveries of the discharges are rarely less than 50 per cent., while of the cases remaining not more than from 10 to 20 per cent. can be considered curable. The "remaining" cases in large hospitals, which have been some years in operation, may be considered as representing the incurables, and as furnishing their principal annual mortality. A table, shewing the per-centage of recoveries at several asylums, proves that the per-centages calculated on the admissions do not bear any definite proportions to those calculated on the discharges.

"It is sufficient that we should be aware that the true per-centage of recoveries is one which, in different institutions, in different degrees, is intermediate to that calculated on the admissions and that calculated on the discharges. While, therefore, I freely admit that the plan of calculating them on the admissions fails in exhibiting with precise accuracy the results of treatment in any hospital for the insane, I yet believe that it affords a much nearer approximation to the truth, when considerable periods are concerned, than that calculated on the discharges; and that therefore it is very decidedly to be preferred to the latter method."

Another table is given, shewing that, in the asylums at Lancaster, Glasgow, and Hanwell, and the Retreat, the proportion of recoveries calculated on the admissions is least in the infancy of the respective institutions, and increases with their age, the very reverse being observed when the calculation is made on the discharges. After the Lancaster Asylum had been in operation five years, the proportion on the *admissions* was 28·8, and on the *discharges* 52·7; but at the end of 26 years the proportion on the *admissions* was 40·3, and on the *discharges* 48·1. At the Retreat, at the end of five years, it was 26·1 on the admissions, and 62·1 on the discharges; while at the end of 45 years these figures were 47·8 and 55·6.

Mr. Farr has exposed the fallacy of calculating the *mortality* upon the *admissions*, as practised by Esquirol, Burrowes, and most other authors. The proper method consists in taking the mean annual per-centage of deaths calculated on the *average number resident;* but unfortunately few asylum reports furnishing aggregate results state their mean numbers resident or average population. A table is given, in which the mortality is calculated in both these modes. From this we find that the *mean annual mortality per cent. of residents* was 10·75 at Hanwell; 7·45 at Nottingham; 10·5 at Bethlem; 4·78 at the Retreat; 13·67 at Lincoln; and 15·54 in the Metropolitan Licensed Asylums; but that, when this was calculated upon the *admissions,* the per-centages of these respective institutions were as follow: 34·7; 12·2: 5: 23·1; 18·5; and 27·9! No safe conclusion can be come to from *observations conducted over a brief period only;* and although, under any circumstances, "the undiscriminating comparison of aggregate results is nearly always very fallacious, yet it is particularly so when these apply to short periods, and especially when such periods are the *first* in the history of the institutions to which they refer."

"From a particular investigation of the statistics of a large number of asylums, both in our own and other countries, I find that the *proportion of recoveries,* in nearly every instance, has gone on materially increasing for a considerable period, often amounting to 30 or even 40 years, after their first establishment. The reason of this is evidently found, as has already been hinted, partly in the large proportion of old cases often admitted upon the first opening of the institution; and partly, though in a less degree, in the circumstance of the recovery, in a certain number of cases, requiring a rather considerable period for its completion. On the other hand, the *mortality* is generally more favorable during the early history of an asylum; and during the first 20 or even 30 years of its operations, as the proportion of recent cases admitted increases, and as the old cases die off, it usually continues to undergo a material increase, which often amounts to 50 or 100 per cent. upon the mortality of the first five years. From the following tables we may, I think, conclude that a period of from 20 to 30, or in the case of a small institution, a still greater number of years must elapse before we are authorized in concluding that the experience of a hospital for the insane at all fairly represents the average results of treatment which either have been, or will be, obtained in it." P. 19.

2. *Modifying Circumstances of the Cases themselves.*—Besides the sources of error derivable from faulty methods, others may depend upon the previous circumstances and character of the cases admitted not having been taken into account. Some of these Dr. Thurnam passes in review.

(a.) *Sex.*—As the result of investigating the returns of numerous asylums, both in this and other countries, Dr. T. found, in all but two, the *proportion of recoveries was greater in women than men.* At Glasgow it was so 4 per cent., at Lancaster 7, at Worcester U.S. 19, at the Retreat 20, at Charenton 23, and at York Asylum 28 per cent. At Hanwell, however, the male recoveries exceeded the female by 5 per cent., and at Bloomingdale U.S. (where patients with delirium tremens are admitted) by 28 per cent. So, too, the *mortality of female lunatics* is much less than that of male. In the general mortality of this country the *excess on the side of the male* does not exceed 5 or 6 per cent.; but in the York Asylum it is 93, and at St. Luke's 96 per cent., or very nearly double. It is 72 at Hanwell, 57 at Glasgow, and 34 at the Retreat. At the Belfast Asylum alone has the mortality of women exceeded (12 per cent.) that of men.

Hence, in estimating the results of treatment at different institutions, we must always remember that these will be less favourable in proportion as the male admissions preponderate.

In connexion with this part of the subject, we may here advert to Dr. Thurnam's Essay (re-printed in another part of this volume) upon "The *Relative Liability of the Two Sexes to Insanity.*" He believes the prevailing opinion, that females are most liable, to be founded upon erroneous data. Esquirol having collected tables from all countries, and finding by them that there were 37 male to 38 female lunatics, hastily concluded that the disease therefore preponderated in the latter. According to the Census of 1841, however, the excess of females of all ages over males is 4 per cent., and about 8 per cent. above 15 or 20 years of age. The excess is as high as 12 per cent. from 20 to 30, and is 6 per cent. from 30 to 40, and 4 per cent. from 40 to 50. Thus, supposing the liability to insanity only *equal*, we ought to have a preponderance of females, especially between the ages of 20 and 50, within which period insanity chiefly occurs for the first time. The only institutions in which a material preponderance of female admissions for considerable periods occurs are St. Luke's and Bethlem, amounting to 20, 30, or even 45 per cent. This may, in some degree, perhaps, be accounted for by peculiarities of the metropolitan population; for while, in the kingdom at large, the excess of females between 20 and 50 is but 8 per cent., it is 18 in the metropolis. The preponderance, however, does not extend to the upper and middle classes, for the male admissions in the licensed private asylums exceeded the female by 38 per cent.

Esquirol has moreover fallen into the fallacy of comparing the *existing* instead of the *occurring* cases, but the mortality of male (taking the asylums generally) exceeds that of female lunatics by 50 per cent.; so that existing cases accumulate much faster in women than in men, and therefore afford no data for judging of the comparative frequency of the disease. Owing, too, to the greater number of relapses which take place in women, the number of admissions is raised higher than the true proportion.

Table No. 15, shews the sex of 67,875 lunatics admitted into various British asylums to 1844. Of these, 47,183 were paupers—24,372 males, 22,811 females: 19,314 were in private asylums—10,980 males, 8334 females; and 1379 were mixed—furnishing 692 males, and 687 females.

"It is indeed highly probable that different countries, and perhaps even the same country at different periods, as well as different communities, and different ranks and classes, in the same country, may vary very much as regards the proportion in which men suffer from insanity more than women. Thus, a comparison of the proportion of males and females of the two classes of pauper and private patients admitted into different classes of asylums, leads to the conclusion that, in this country, a larger proportional number of men become insane in the higher than in the lower ranks of society. This may, however, admit of explanation, if, as is not improbable, women in the humbler ranks of life, who are the subjects of insanity, become sooner pauperized than men under the same circumstances. It also appears tolerably well ascertained that a larger proportion of women, relatively to the other sex, become insane in France than in England; though, as we have seen, this is less certain as regards the metropolis when compared with the rest of this country. In this respect we have seen that the statistics of our own metropolis appear to resemble France rather than those of the rest of England." P. 153.

The experience of the Retreat would at first sight seem to militate against the author's deduction, inasmuch as the number of women admitted has exceeded that of the men by 18 per cent., *i. e.* 45 men have been admitted to 55 women. But, among the Quakers, the females of all ages exceed the males by 20 per cent.; and above 15 years of age the excess reaches 30 or 35 per cent. So that, supposing the experience of the Retreat to represent the proportions among the Quakers at large, there are still from 10 to 14 per cent. more males than females attacked. However, in the various points in which females have the advantage over males they have it less so among the quakers. "This is worthy of notice, as it is probably due to the greater general regularity of life in the men of this community as compared with that of men in the community at large; or, at least, than in those parts of it which furnish inmates to the asylums compared."

(b.) *Age.*—The statistics of lunatic asylums shew that the *probability of recovery* is greatest in the young, and decreases regularly as age advances; but that the *mortality* increases much more rapidly with age than it does in the general population. Dr. Thurnam has an interesting paper upon the "*Liability to Insanity at different Ages.*" He criticizes the conclusion Esquirol and others have arrived at from the examination of numerous tables of *existing* cases, that individuals become more obnoxious to insanity the longer they live after maturity. By examining the ages of 12,575 admissions into British Asylums, it will be found the greatest number have occurred between the ages of 30 and 40; but the period at which a patient is first brought to an asylum is a very uncertain indication of that at which the attack of insanity first came on. By comparing the ages of *occurring* cases at Hanwell (1840-4) with the numbers living at corresponding ages in England and Wales, Dr. Thurnam arrives at the conclusion that the period of life most liable to insanity is from 20 to 50 or 60. From 30 to 40 the liability is usually greatest, and it decreases in each succeeding decimal period. So far from the liability being greatest in old age, it is nearly twice as great from 30 to 40 as from 50 to 60, and much more than twice as great than at any age subsequent to 60. At the Retreat the ages between 20 and 30 are those most liable.

(c.) *Rank, prior habits, &c.*—The prior condition of the patient in this, as in other diseases, much influences the probability of recovery and amount of mortality. The Retreat is an establishment for persons of a select and favourable class. The recoveries there have been at the rate of about 50 per cent. and the mortality is but 4·7 per cent., while at the Wakefield asylum the recoveries are 43·6 and the mortality 15·7. At Lancaster, the recoveries are but 39·8. The mortality of the Metropolitan Licensed Pauper Asylums is 20·68 per cent., while in those for private patients it is but 10·94. In all these cases other circumstances may doubtless exist for the explanation of the discrepancy; but it is obvious that, in comparing the results derived from one asylum with those obtained at another, we must especially bear in mind the condition of society of their respective populations.

(d.) *Causes of Disease.*—Dr. Thurnam observes that too great carelessness has occurred in many institutions in registering assigned causes, and in drawing inferences from numerical results thus obtained. For our parts we see almost insuperable difficulties in obtaining acurate details on this head, and quite agree with the following opinion of Dr. Conolly: " With all our care, a true account of the causes and commencement of the malady is so difficult to be obtained, that I look with much incredulity on all statistical returns that have been made in this or any other asylum as relates to these particulars."

(e.) *Imperfect Classification and Specification of the Forms of the disorder* have also led to erroneous conclusions.

(f.) *Duration of the Disorder.*—That the *recoveries* are frequent in proportion as the cases are *recent* is well known, and of vital importance it is that it should be still better known. Dr. Thurnam divides the consideration of this section of his subject into two parts, the *duration of the disorder on admission,* and the duration of the *treatment or residence.* At the Retreat, the probability of Recovery in cases admitted within the first 3 months, is 4 to 1, and when uncomplicated with serious bodily disorder 9 to 1: while in cases not admitted until after 12 months, it is less than 1 to 4. When the cases are separated into only two categories, viz. those in which the disease is of less, and those in which it is of greater duration than 12 months, the difference in the amount of recoveries is very striking. Thus, we find in the first of these classes they were 49·26 upon the admissions at Maidstone; 50·95 at Lincoln, and 61·87 at the Retreat; while, for the other class they were but 4·84, 9·62, and 18·88. The mortality is, however, greater in recent cases, for at the Retreat its mean annual amount during 48 years has been 7·3 per cent. in cases in which the disease has existed but 3 months, and but 4·57 when it has existed more than 12 months. In comparing the experience of different institutions, we must not neglect to take into account the *duration of residence or treatment,* which must materially influence the amount of recoveries and deaths. Thus, in some, discharges are compulsory within a twelvemonth, and, in others, the expense leads to the withdrawal of patients. At the Retreat, cases are allowed to remain an unlimited period, nor are they hurried away too early in convalescence: and its large proportion of cures and low degree of mortality may be in some degree due to this prolonged residence. One third of the entire recoveries occurred after the first year, and nearly one-sixth after the second.

Dr. Thurnam concludes this part of his subject with some directions for collecting and registering the Statistics of the Insane, which are well worthy of notice; and indeed all who contemplate the contribution of additional facts concerning the various conditions of the insane will do well to peruse the observations of this cautious and able writer.

### Management of the Insane.

Dr. Thurnam passes in review the various particulars of the treatment of the insane capable of influencing the statistical results, and cites the experience of the York Asylum prior to its reform, in proof of the extent of the operation of these. He also presents tabular views of the results of treatment in several of the principal British and foreign asylums. Into these details we cannot follow him ; but may refer to a few observations, and collate them with remarks on the same topics contained in some of the various Asylum Reports* before us.

1. *Exercises, Occupations, and Amusements.*—The important agency of these has now been long established, and most of the recent improvements have consisted in carrying them still farther into operation than heretofore. Open air exercise is best of all, and the Irish district asylums are now usually furnished with farms of 20 acres and upwards. The miserable inadequacy in this respect of several of the establishments in the metropolis and other large towns is to be much deplored. Dr. Thurnam states that the amount of labour he saw performed in many Scotch and some Irish asylums was surprising; and there may obviously be an extreme sometimes in his direction. There is more difficulty in finding employment for the men of the more opulent classes, and at the Retreat there is always a larger proportion of females than males engaged. The officers of the Lancaster asylum speak of the great advantages which have attended the increase of the sphere of occupation during the last few years. Of 325 inmates 195 there followed husbandry or some trade occasionally, and above 100 might be seen actively employed daily. In this establishment too, much attention is also paid to amusements, such as country excursions, evening parties, games, keeping animals and plants, engravings and books, &c. Dr. Brigham has seen immense advantage derived from a fancy fair, and the consequent erection of a green-house. He encourages music and dancing, but not amusements which require the mixing of the sexes.

2. *Government and Attendants.*—Dr. Thurnam, as indeed nearly every one who has written on the subject, insists upon the great superiority of a *resident* to a visiting physician : and if there is one point more important than another in the management of an asylum it is the entrusting to this resident officer the supreme and undivided controul over the whole of the inmates, sane and insane. Even matrons are frequently mischievous officers from their officious intermeddling. The proportion of attendants to patients is very various in different establishments. Thus, at Wakefield it is but one to 22, at Hanwell to 18. In the Irish district asylums it is 1 to 13, or with assistants 1 to 9. At Lincoln it is 1 to 9. In the Scotch asylums 1 to 10. At Sieburg, according to Jacobi, it is 1 to 7 or 8; and at the Retreat it is 1 to 6 or 7. In establishments for persons in the easy classes of society the proportion should not be less than this last; but in the pauper establishments where the patients often assist each other, it need not be more than 1 to 12 or 15. Dr. Brigham states that the proportion in the State Asylum of New York, is about 1 to 10. Both he and the officers of the Lancaster Asylum lay great stress deservedly upon the

* We shall feel obliged by our friends in various parts continuing to forward us these documents. Even when not requiring special comment they are often of great use in aiding the formation of correct opinions.

employment of efficient and respectable persons; and the latter have endeavoured to instruct the attendants in the nature of the duties required of them, and have considerately so arranged that they may have opportunities of obtaining sufficient relaxation. There cannot be a doubt that the nurturing a respectable body of officials of this kind is of the last importance. Their non-existence has thrown, and will yet throw, obstacles in the way of rapid improvement. Their education, comfort, and due relaxation are well-earned by their arduous and important duties.

3. *Clothing.*—As a general rule, the insane require warmer clothing than the healthy, especially as regards their feet, the circulation being often feeble, and the functions of the skin imperfect. We must not wait for their expressing their sensations, which they often will not or cannot do, or these may not exist. The suffering from cold was one of the worst enormities of bye-gone management, and Dr. T. states that, even now, he occasionally witnesses patients in large asylums without shoes and stockings, or a sufficiency of other garments. "It is very desirable," says Dr. Brigham in his Report, "that extra and better garments should be sent with those accustomed to them, that when they become better, and when they walk or ride out, attend religious worship, &c., their self-respect may be preserved. This is important, and should not be neglected."

4. *Diet.*—Dr. Thurnam treats upon this at considerable length, but we have space for but a few remarks. He justly observes that a liberal, nutritious, but simple diet, is that upon which patients thrive, and most recoveries occur. He thinks the diet should approximate to that habitually employed by the middle classes, thus raising that of the pauper, and rendering that of the wealthy man somewhat plainer. He believes that the dietaries of some of the pauper asylums contain too little animal food. He gives a diet table of seven asylums in which great discrepancies exist: and states that, in three of those in which the better diet prevailed, the recoveries were 43·7 per cent., and the mortality 9·35: while in the others, where a poorer diet existed, the recoveries were only 36·75 per cent., but the mortality was 14·54. Other causes may have also contributed to produce this result. Dr. Brigham employs only the best food, which is served up in crockery (at Lancaster the patients, too, derived great satisfaction for the substitution of this for wooden trenchers), and furnished to the inmates as if they were boarders and not patients. As a general rule he does not limit the amount eaten. "The insane require full as much food as the sane, and we think rather more; many of them have been reduced by sickness or by their real or imaginary troubles, before they came under our care, and when they begin to recover eat very heartily. The total increase in weight of the 132 discharged last year was 1565 pounds."

5. *Pharmaceutical Treatment.*—Dr. Thurnam observes that, although he agrees with those who proscribe lowering treatment as a general rule, and approve of a supporting regimen, he is far from discountenancing moderate local depletion and other antiphlogistic means in appropriate cases. The overlooking bodily disorder in insanity, and the treating the disease by mere routine of bleeding, purgatives, or emetics, are both erroneous extremes.

6. *Restraint.*—The practicability of abolishing all personal restraints has been finally established by Dr. Conolly's grand experiment at Hanwell, so lucidly and minutely described in his Lectures. How Dr. Stewart, of the Belfast Asylum, can state "that total abolition of restraint appears to be only one of the many vulgar delusions and speciously popular *ad captandums* of the day," we cannot imagine. From the epoch of the institution of the courageous experiment may be dated a vast progress in the improved condition of the insane; for, if its complete imitation could not be always realized, it at least exhibited to all the shallow pretexts upon which excessive restraints rested, and put their much longer continuance out of the question. The consequence now is, that, in every well-conducted asylum, cases requiring restraint of any kind have become wonderfully few, shewing how much groundless fears and harsh treatment had multiplied them heretofore. Taking Dr. Conolly's explanations, however, as we find them in his Lectures, in which he complains that his mode of employing seclusion has been misrepresented, we must still retain the opinion formerly expressed, that the restraining the few patients who yet require it by means of slight bands, as practised by Drs. Brigham, Thurnam, and others, is preferable to seizure by numerous attendants and seclusion. Of course the most vigilant care is supposed to be taken that the restraint shall be discontinued as soon as possible, and that it shall never be resorted to, save by the sanction of the physician. Happily, however, cases requiring either restraint or seclusion are becoming fewer and fewer; and it is cheering to find, from the Report of the officers of the Lancaster Asylum, that the accommodations of that edifice have been increased, at even the expense of its apparent security, with the best effects.

"As illustrative of the effects produced on the insane by the removal of obstacles to freedom, or such as suggest a feeling of confinement, it may be remarked, that the attempts to escape from the wards were diminished after the facilities of escape were greatly increased. In 1826 an addition was made to the building, called the criminal wing, constructed in every way so as to offer most formidable obstacles to escape, by means of strong dark cells and iron gates. It is somewhat remarkable, that the only unrecovered patients who have escaped from the building since its establishment, have made their exit from this portion especially adapted for their security."

7. *Religion.*—Dr. Thurnam, in common with most other principals of asylums, testifies to the great benefit which accrues to many patients from participation in religious worship; and he offers some very suitable advice to ministers upon the mode of performing this delicate duty, and the classes of subjects best suited for commenting upon to the insane. It is obvious here that every thing depends upon discretion, and that a rash, over-zealous man may do immense mischief; and, indeed, there can be no safety in the employment of any such agency, unless it is implicitly submitted to the control and direction of the physician. In an article upon this subject in the "American Journal of Insanity," Jacobi's advice, that the chaplain should render himself familiar with writings on insanity, is reiterated, and a knowledge of anatomy and physiology recommended; but we doubt the soundness of the recommendation, as a smattering of this sort is more likely to beget a self-sufficiency and perhaps contrariety of opinion, adverse to an entire submission to the views and directions of the medical officer. The mischief which an injudicious or fanatical chaplain may do may be in some degree judged of by the following passage from a letter written by the chaplain of the Virginia Asylum, U. S., and quoted (with disapprobation) in the journal just referred to. "If the insane can be made to comprehend religious instruction, why may they not become interested in their spiritual condition? And if they should become as deeply interested in their spiritual welfare, as we have known some of the entirely sane to do through the instrumentality of religious instruction, why may not *this religious concern operate on the principle of revulsion in effecting their cure?*" Dr. Thurnam observes that the services should not exceed one hour; but we think half that time, exclusive of the singing, should suffice.

8. *Schools.*—It is a disgrace to our English Asylums that these have not been established within their walls. Their success in Paris has been recently and graphically described by Dr. Conolly, and Dr. Brigham thus speaks of those established in the New York Asylum.

"Our confidence in their utility, and even their necessity for the improvement of many of the insane, has increased. We find the patients who have recovered there, look back to their attendance in school as the greatest enjoyment they had, and often allude to it, and to the advantages they derived from it, in their letters. A manual or book of instruction, containing short precepts and maxims for the guidance of the insane, respecting the preservation of health—the government of the passions and control of the feelings, portions of which they would commit to memory, we believe would do much good, and we hope to be able to prepare such in the course of another year. Those who have been once insane are so liable to a recurrence of insanity, that we feel as if we had not done a patient all the good we ought by curing him of one attack, but that we should endeavour so to instruct him that he may prevent another. We have great confidence, in many instances, of *man's power over himself to prevent and control insanity.* But to accomplish this many need to be enlightened."

The present article has run over a larger space than we intended it to have occupied; but we trust that some of the topics adverted to in it will not prove devoid of interest at a period when we are upon the eve of a great change, and we believe improvement, in the provisions for lunatics in this country. We are not sufficiently acquainted with those intended for Ireland and Scotland to be enabled to offer an opinion concerning them. The existing district asylums of the former country are described by Dr. Thurnam and others as being well-conducted establishments, so that their multiplication is very desirable. In Scotland much, very much, remains to be done to place the condition of lunatics, and indeed paupers in general, upon a satisfactory footing. Dr. Conolly has recently exhibited the general excellent management of the large, indeed far too large, asylums of Paris; and the accounts we receive of those of the United States*

---

* We are not aware of the exact present provision for the insane in America.

are even more satisfactory. Indeed, it is cheering to find the cause of humanity progressing on every side. Enlightened observers are now engaged in all parts of Europe and America in contriving ameliorations, superintending their execution, and recording the results ; so that, ere long, vast bodies of valuable and authentic information will have become accumulated. The medical directors of public insane establishments seem to be free from the jealousies and rivalries which sometimes impede the usefulness of our profession, a honorable emulation and courteous co-operation stimulating and augmenting their power of benefiting those committed to their charge. With the increased numbers of these officers consequent upon the erection of the new asylums, it is very desirable that some plan for the uniform preparation and exchange of Reports and for occasional meetings should be organized. An Association has been formed for such purposes in the United States, and we believe upon a small scale among ourselves. In the former country, too, a cheap quarterly periodical, " The Journal of Insanity," has been established by Dr. Brigham, of which, judging from the few numbers which have irregularly reached us, we can report favourably. How far it would be expedient to establish some such a medium of intercommunication here is perhaps doubtful, when the abundant facilities offered by existing periodicals are considered.

In 1843, however, Dr. Pliny Earle stated, in the American Journal of Medical Science, that there were 20 public asylums, providing 2647 beds, and possessing 671 acres of ground.

